# Tunneling nanotubes contribute to Zika virus pathogenesis at the maternal-fetal interface *in vivo*

**DOI:** 10.1128/mbio.01562-26

**Published:** 2026-08-21

**Authors:** Rafael Tomoya Michita, Long B. Tran, Joyce Jose, Indira U. Mysorekar

**Affiliations:** 1 Department of Medicine, Section of Infectious Diseases, Baylor College of Medicine 171841, Houston, Texas, USA; 2 Department of Biochemistry and Molecular Biology, The Pennsylvania State University 219262, University Park, Pennsylvania, USA; 3 The Huck Institutes of Life Sciences, The Pennsylvania State University 124474, University Park, Pennsylvania, USA; 4 Department of Molecular Virology and Microbiology, Baylor College of Medicine 189531, Houston, Texas, USA; Tsinghua University, Beijing, China

**Keywords:** placenta, IFN-λ, NS1, ZIKV, TNT

## Abstract

Zika virus (ZIKV) infection during pregnancy can result in severe fetal outcomes, yet the mechanisms of transplacental dissemination remain incompletely defined. We previously showed that ZIKV induces tunneling nanotubes (TNTs), actin-rich intercellular conduits that enable direct cell-to-cell transfer of viral components. Here, we investigated the *in vivo* role of TNTs in ZIKV maternal-fetal transmission using complementary pregnancy models. A TNT-deficient ZIKV mutant (ZIKV^ΔTNT^), harboring a change between residues 40 and 52 of the nonstructural protein 1 (NS1), showed markedly reduced viral dissemination to maternal and fetal tissues across all models tested, whereas the TNT-competent ZIKV established a robust infection. ZIKV^ΔTNT^ infection was associated with reduced placental pathology, altered junctional-to-labyrinth architecture, improved placental efficiency, and protection from fetal growth restriction. Loss of TNT-forming capacity also limited viral persistence despite maternal type III interferon (IFN-λ) responses, suggesting a role for TNTs in immune evasion. Together, these findings provide *in vivo* evidence that TNTs are a key mechanism by which ZIKV enhances dissemination, promotes placental dysfunction, and drives fetal pathogenesis.

**IMPORTANCE:**

Zika virus infection during pregnancy can cause severe fetal abnormalities, yet how the virus overcomes the placenta remains incompletely understood. Here, we show that ZIKV exploits direct intercellular connections called tunneling nanotubes (TNTs) to facilitate cell-to-cell transmission and impact placental infection and fetal outcomes. Using multiple pregnancy models, we demonstrate that viruses capable of forming these structures disseminate more efficiently, damage the placenta, and lead to fetal growth restriction, whereas TNT-deficient viruses show reduced infection and milder disease. Importantly, TNT-mediated dissemination is associated with viral persistence despite maternal interferon responses, suggesting a role in immune evasion. These findings identify TNTs as a previously underappreciated pathway of viral transmission during pregnancy and suggest new therapeutic targets. More broadly, TNTs may contribute to the pathogenesis of other vertically transmitted or emerging viral infections.

## INTRODUCTION

Zika virus (ZIKV) is a mosquito-borne *Orthoflavivirus*, belonging to the *Flaviviridae* family. ZIKV is unique among members of this group because it can be transmitted not only by mosquitoes but also through vertical and sexual routes (1, 2). Infection during pregnancy is associated with intrauterine growth restriction, fetal demise, and congenital Zika syndrome, including microcephaly (3–5).

Vertical transmission of ZIKV can occur via transplacental, intrauterine, or transvaginal routes (6–10). Although multiple mechanisms have been proposed to explain how ZIKV traverses the placenta, including receptor-mediated entry, paracellular passage, and extracellular vesicle-mediated transcytosis (11, 12), these pathways do not fully account for viral dissemination. Consistent with this, receptor-deficient mouse models, including those lacking TAM (13) or HAVCR1 (12, 14) receptors, remain susceptible to ZIKV infection during pregnancy, highlighting the existence of alternative, receptor-independent mechanisms of viral entry. Despite this, the placenta remains an effective barrier that protects the fetus from ZIKV infection, owing in part to its robust antiviral interferon (IFN) responses (15–17).

Recently, our group identified a novel mechanism that ZIKV can co-opt to enter multiple cell types at the placenta through tunneling nanotubes (TNTs) (18, 19). TNTs are actin-rich conduits that allow for cytoplasmic continuity and direct transport of cargo, including organelles, nucleic acids, proteins, lipids, and signaling molecules (20, 21). TNT-like structures have been detected *in vivo* across multiple tissues and model systems (22–30), supporting their role as a conserved mechanism of intercellular communication. Importantly, TNTs have been found in the human placenta mediating intercellular connections between endothelial cells, although their functional roles remain to be defined (31). More broadly, TNTs have been implicated in intercellular communication in cancer, tissue injury, development, stress, and infection (32, 33).

We further demonstrated that the ZIKV nonstructural protein 1 (NS1) uniquely induces TNT formation and that amino acids 40–52 within its wing domain are critical for this activity, and mutations in this region abrogate TNT formation (18). Consistent with this, ZIKV infection induces TNT formation, promoting intercellular trafficking of viral particles and proteins between placental cells while facilitating evasion of antiviral IFN responses. In contrast, infection with a TNT-deficient ZIKV mutant (ZIKV^ΔTNT^) elicits a robust antiviral IFN-λ1/2/3 response compared to wild-type virus. This heightened antiviral response restricts viral transmission, suggesting that TNT-mediated trafficking enables cell-to-cell transmission while evading host defenses.

However, whether TNTs contribute to ZIKV dissemination *in vivo* and how they impact placental function and fetal outcomes remain unknown. Here, using complementary pregnancy models of infection and a TNT-deficient ZIKV mutant, we demonstrate that TNT-associated viral functions promote placental-fetal infection, placental pathology, and adverse fetal outcomes.

## RESULTS

### TNT-forming capacity of ZIKV increases placental pathogenesis and fetal adverse outcomes

**Fig 1:**
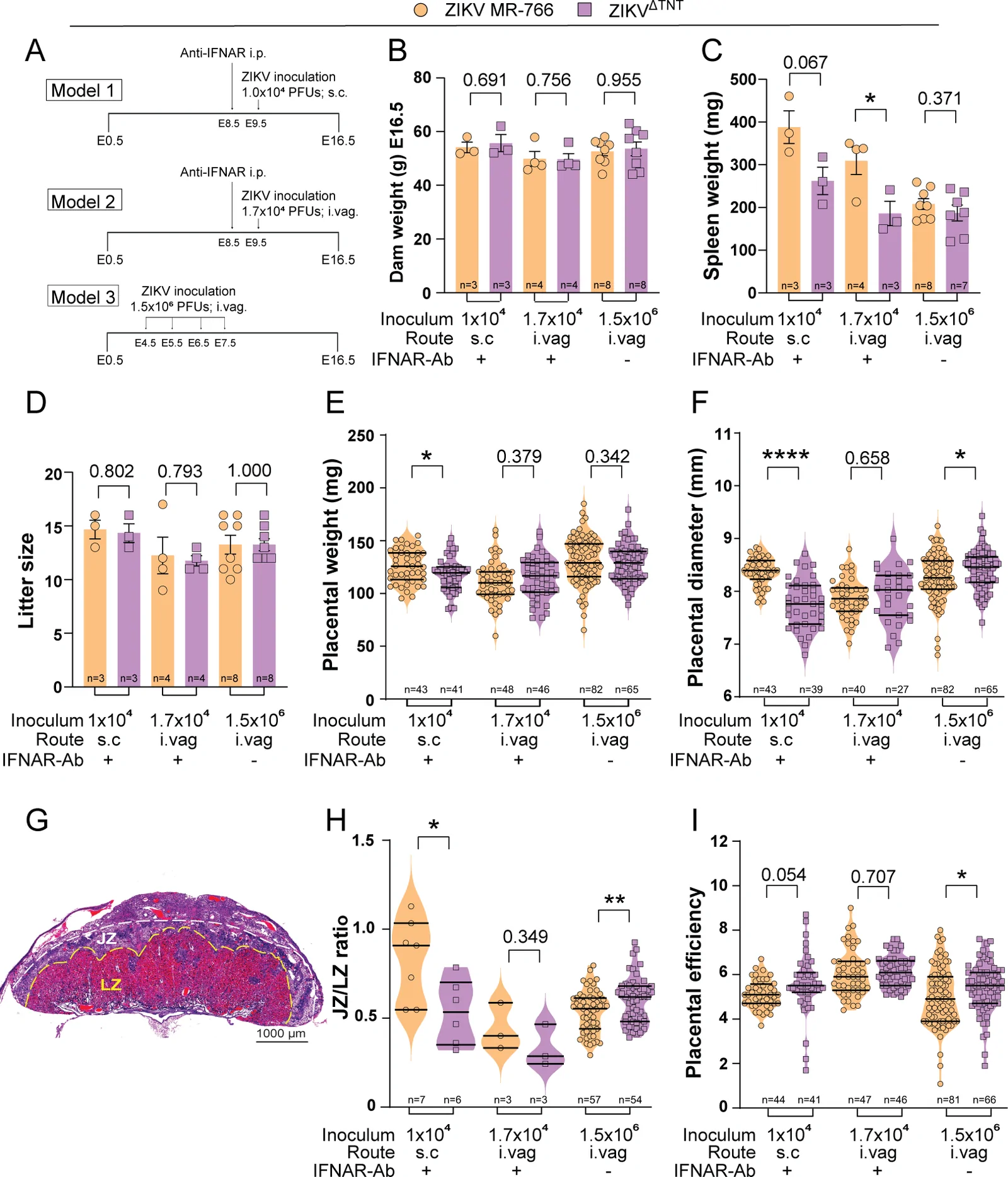
Loss of TNT-forming ability of ZIKV ameliorates placental pathogenesis *in vivo*. (**A**) Schematic of pregnant mouse infection models. CD-1 pregnant females (3–4 months old) were treated with intraperitoneal Ifnar1 antibody (IFNAR-Ab) at E8.5 and inoculated with ZIKV (MR-766 or ZIKV^ΔTNT^) at E9.5 via subcutaneous (s.c.; model 1, 1 × 10⁴ PFU) or intravaginal (i.vag; model 2, 1.7 × 10⁴ PFU) routes. Immunocompetent pregnant mice were inoculated intravaginally at E4.5–E7.5 (model 3, 1 × 10⁶ PFU). Dams were monitored daily and euthanized at E16.5. In all models, dams were monitored daily for health conditions until euthanasia at E16.5. Maternal infection burden at E16.5 is represented as (**B**) body weight (g), (**C**) spleen weight (mg), and (**D**) litter size. Placental infection outcomes were determined at E16.5 by measuring the (**E**) weight (mg), (**F**) diameter (mm), and (**G and H**) junctional zone (JZ) to labyrinth zone (LZ) area ratio. (**G**) Representative H&E image delineating the JZ and LZ regions. (**I**) Placental efficiency calculated as fetal-to-placental weight ratio. Data are presented as mean ± SEM (**B–D**) or median with interquartile range (25th–75th percentiles) (**E, F, H, I**). Sample size (*n*) is indicated below each graph. Statistical significance was determined by an unpaired *t*-test with Welch’s correction; *P ≤ 0.05, **P ≤ 0.01.

We previously demonstrated that loss of TNT-forming capacity of ZIKV (ZIKV^ΔTNT^) impairs infection and intercellular transmission of ZIKV in placental trophoblasts (18). Here, we investigated the role of TNTs in facilitating ZIKV maternal-fetal transmission and pathogenesis *in vivo* using pregnancy models accounting for gestational timing (early and late placentation), route of transmission (subcutaneous and intravaginal/sexual), intraspecific genetic diversity (outbred mouse strain), and antiviral IFN response (transient Ifnar1 blockage) (Fig. 1A).

In all models tested, ZIKV challenge did not result in mortality and had minimal impact on maternal weight gain, overt clinical disease, or pregnancy outcomes, irrespective of route of infection, virus inoculum, or immune status at the time of infection (Fig. 1B and D). However, Ifnar1 antibody-treated dams infected with the TNT-deficient ZIKV^ΔTNT^ exhibited reduced spleen weight (Fig. 1C), suggesting reduced systemic immune activation and viral dissemination. No differences in fetal resorption rates were observed between experimental models (data not shown).

Next, we examined whether the TNT-forming capacity of ZIKV contributes to placental pathogenesis. In Ifnar1 antibody-treated dams, infection with TNT-deficient ZIKV^ΔTNT^ resulted in significantly improved placental outcomes compared with TNT-competent ZIKV, as demonstrated by reduced placental weight and diameter (Fig. 1E and F, model 1). These observations are consistent with prior reports of ZIKV-associated placental hyperplasia and vascular disruption (34–36). Also, TNT-competent ZIKV infection resulted in elevated junctional zone (JZ)-to-labyrinth zone (LZ) ratios (Fig. 1H, model 1) and a trend toward reduced placental efficiency (Fig. 1I, model 1), compared with ZIKV^ΔTNT^. It is noteworthy that these differences were not observed in dams infected intravaginally under similar conditions, including viral load (1.7 × 10^4^ PFU), immune status (Ifnar1-antibody treatment), and gestational stage (E9.5), suggesting route-dependent TNT differences in viral infection and pathogenesis during pregnancy (Fig. 1E through I, model 2). Of note, exploratory analyses showed that both TNT-competent ZIKV and ZIKV^ΔTNT^ subcutaneous infection affect placental outcomes compared with uninfected controls, suggesting that TNT deficiency attenuates but does not fully rescue ZIKV-associated placental alterations (Table S1, model 1).

To further investigate route-dependent TNT differences in viral infection and pathogenesis during pregnancy, we leveraged a model of intravaginal exposure during early gestation (E4.5 to E7.5), a window known to be permissive to ZIKV infection (8). Because TNTs facilitate direct cell-to-cell viral dissemination and may allow infected cells to sustain local viral spread at the vaginal mucosa despite immune-mediated clearance of cell-free virus, we tested whether sustained viral exposure across a susceptible gestational window enhances TNT-dependent ZIKV infection at this site. To this end, immunocompetent pregnant dams were challenged with ZIKV over four consecutive days (Fig. 1A, model 3). We found that TNT-competent ZIKV infection resulted in decreased placental size (diameter), JZ/LZ ratios, and placental efficiency (Fig. 1F through I, model 3), suggesting that TNTs contribute to ZIKV-associated placental pathogenesis. Of note, differences in immune status, route of infection, and exposure regimen may account for the model-dependent directionality of placental outcomes across models. In this context, E4.5–E7.5 spans a critical window of embryonic development and early placentation, making comparisons between infection models challenging. Nevertheless, the increased placental size, JZ/LZ ratio, and placental efficiency observed in the ZIKV^ΔTNT^ group suggest reduced placental pathogenesis compared with placentas from TNT-competent ZIKV-infected dams.

**Fig 2:**
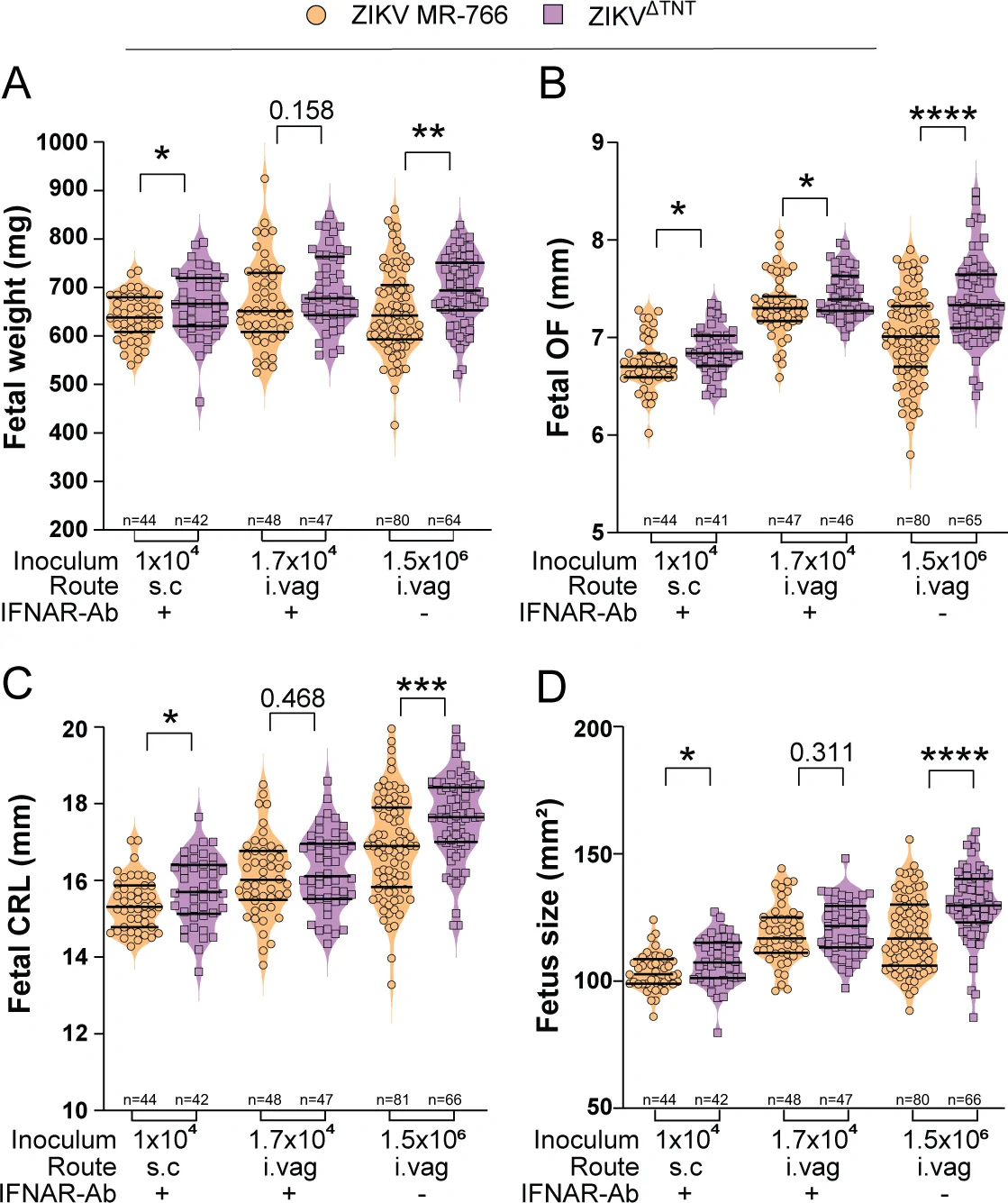
ZIKV^∆TNT^ fails to cause fetal adverse outcomes *in vivo*. Fetal outcomes from pregnant dams infected with ZIKV (MR-766 or ZIKV^ΔTNT^) were assessed at E16.5 and stratified by infection model. (**A**) Fetal weight (mg), (**B**) occipitofrontal (OF) distance (mm), (**C**) crown-to-rump length (CRL; mm), and (**D**) fetal size (OF × CRL; mm²). Median with interquartile range (25th–75th percentiles). The total number of fetuses (*n*) is indicated below each graph. Statistical significance was determined by an unpaired *t*-test with Welch’s correction. *P ≤ 0.05, **P ≤ 0.01, ***P ≤ 0.001, ****P ≤ 0.0001.

We next evaluated the impact of ZIKV TNT-forming capacity on driving adverse fetal outcomes (Fig. 2A through D). In infection models 1 and 3, we consistently found that fetuses from dams infected with ZIKV^ΔTNT^ exhibited increased fetal weight (Fig. 2A, models 1 and 3), and overall growth as measured by fetal occipital-frontal (OF) length (Fig. 2B, models 1 and 3), crown-to-rump length (CRL) (Fig. 2C, models 1 and 3), and fetal size (OF × CRL) (Fig. 2D, models 1 and 3).

Of note, fetal outcomes resembled the observed placental alterations across infection models. In contrast, despite no placental changes in model 2, we found increased OF length in ZIKV^ΔTNT^-exposed fetuses (Fig. 2B, model 2). Our data suggest that fetuses from dams infected with TNT-competent ZIKV show significant fetal growth restriction compared with ZIKV^ΔTNT^ (Fig. 2A through D, models 1 and 3). Furthermore, these findings support that TNT-mediated viral dissemination has minimal impact on maternal disease but drives placental pathology, which in turn is associated with adverse fetal outcomes.

### TNT-forming capacity enables ZIKV placental infection and persistence despite maternal interferon responses *in vivo*

**Fig 3:**
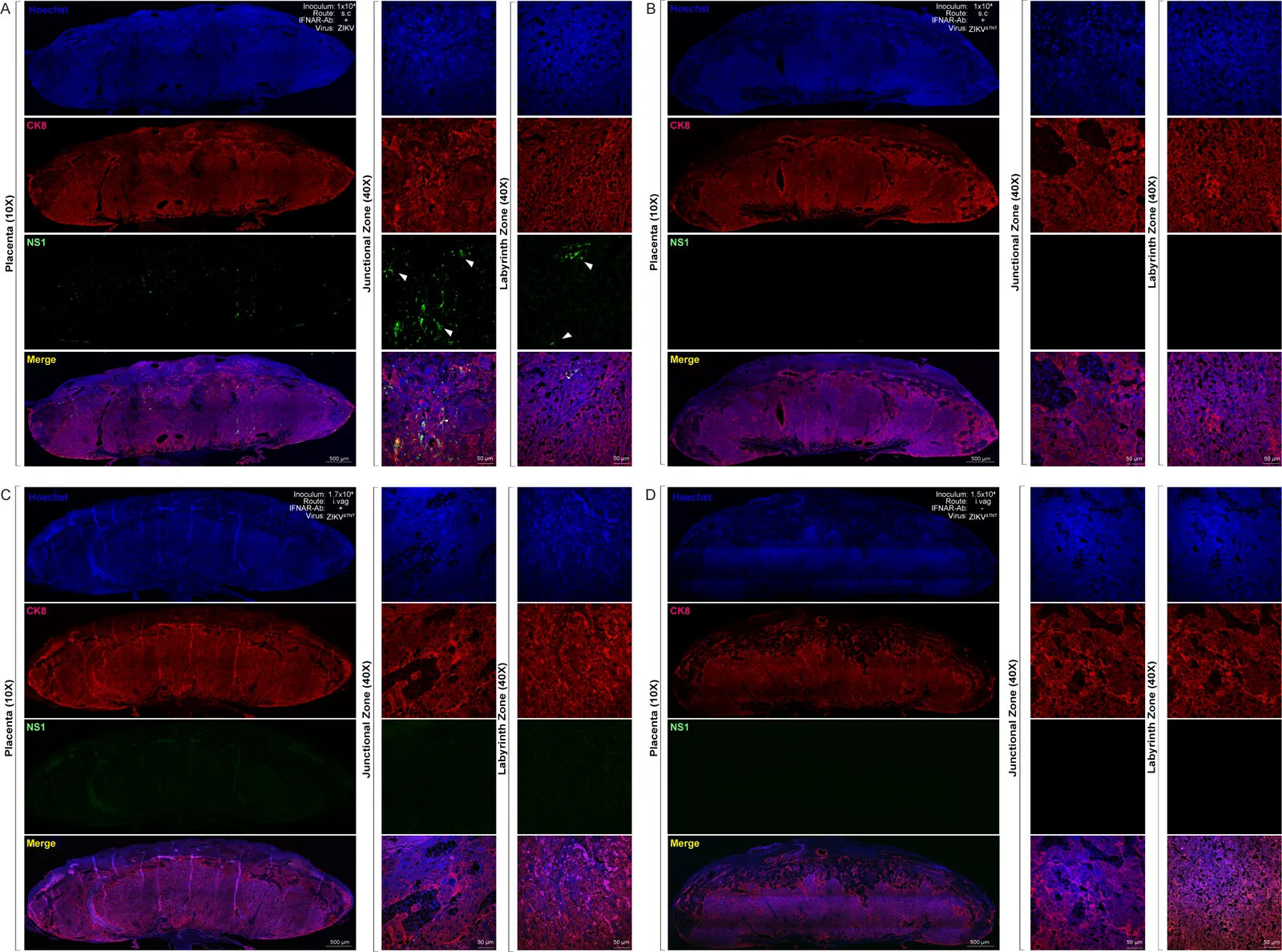
TNT formation is required for efficient placental infection by ZIKV *in vivo*. (**A–D**) Representative immunofluorescence images of E16.5 placentas showing differences in infection between TNT-competent ZIKV and ZIKV^ΔTNT^ across three experimental models. (**A**) Placenta from a dam infected with ZIKV MR-766 in model 1. (**B–D**) Placentas from dams infected with ZIKV^ΔTNT^ in models 1, 2, and 3, respectively. ZIKV-infected dam Placentas were stained for ZIKV NS1 (green), cytokeratin 8 (CK8; red), and nuclei (Hoechst, blue). White arrows indicate NS1 signal, with predominant localization in the junctional zone. Large-area IF images were acquired using a 10× confocal objective (scale bar, 500 µm); insets show representative 40× images of the junctional and labyrinth zones (scale bar, 25 µm). i.vag, intravaginal; s.c., subcutaneous; IFNAR-Ab, Ifnar1 antibody treatment.

**Fig 4:**
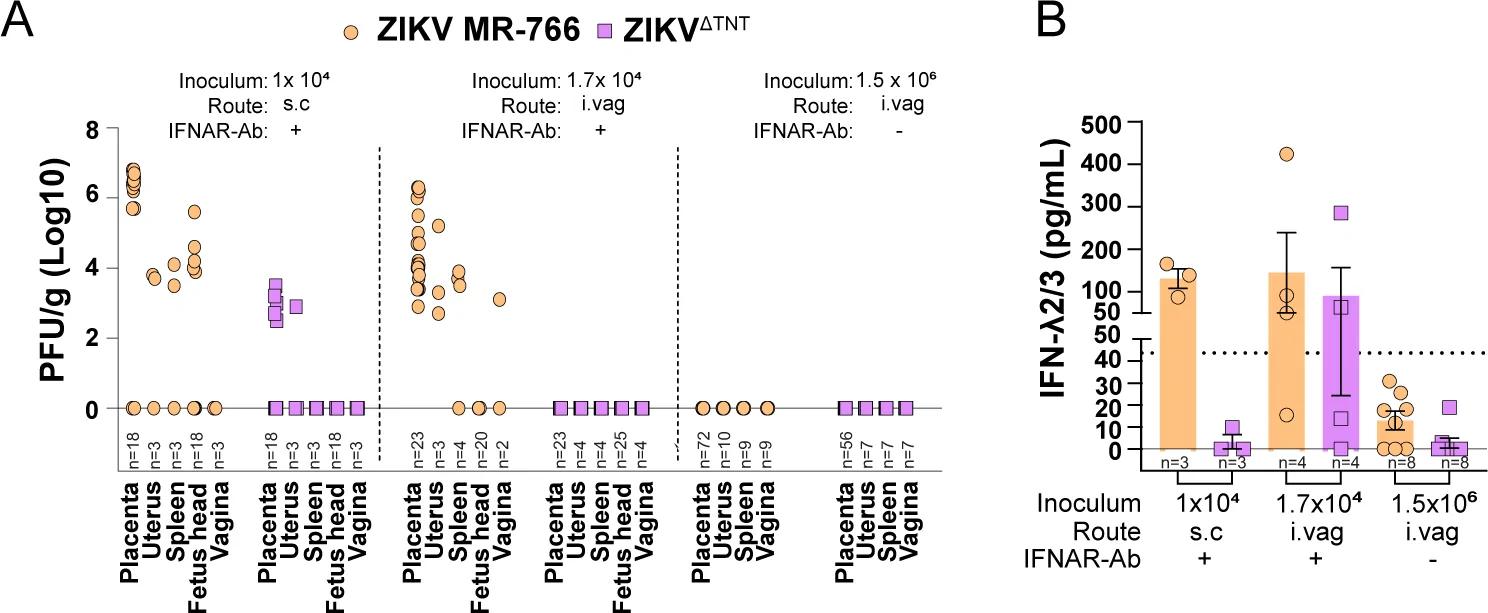
Loss of TNT-forming capacity in ZIKV restricts placental infection and enhances maternal innate immune response *in vivo*. (**A**) Viral infection and dissemination in maternal and fetal tissues across models. Viral titers in tissue lysates were quantified by plaque assay in Vero E6 cells, normalized to tissue weight (g), and expressed as log10 PFU/g. (**B**) Maternal plasma IFN-λ2/3 levels (pg/mL) measured by ELISA and shown as mean ± SEM. The dotted line indicates the assay limit of detection (LOD; 43.71 pg/mL). Sample size (*n*) is indicated below each graph.

To determine whether TNTs facilitate ZIKV infection of the placenta, we examined placentas from infection models for the presence of TNT-forming ZIKV-NS1 protein. Consistent with our previous findings (7), infection of Ifnar1-treated dams with TNT-competent ZIKV led to robust placental infection, with NS1 detected in the junctional and/or labyrinth zones following both subcutaneous and intravaginal challenge (3 of 3 placentae positive for NS1 per route; Fig. 3A; Fig. S1). In contrast, NS1 was not detected in placentae from dams infected with ZIKV^ΔTNT^, despite recovery of infectious virus from placentae in model 1 (Fig. 4A).

Following intravaginal infection of Ifnar1-treated dams, despite the absence of overt placental pathology (Fig. 1E through I, model 2), ZIKV-NS1 was detectable but largely restricted to the junctional zone (Fig. S1). These differences in NS1 distribution likely reflect site-specific efficiency of viral infection (i.e., mucosal or epithelial compartment). In agreement with this hypothesis, higher viral titers of TNT-competent ZIKV were found in maternal and fetal tissues from Ifnar1-treated dams compared with low or undetectable ZIKV^ΔTNT^ titers (Fig. 4A, models 1 and 2). Of note, ZIKV-NS1 was not detected in placentae from immunocompetent dams infected intravaginally irrespective of ZIKV TNT-forming capacity (Fig. 4A, model 3), although transient infection and clearance prior to the experimental endpoint cannot be excluded (8).

We next investigated whether maternal IFN-λ2/3 responses restrict ZIKV infection and whether TNT formation enables viral persistence despite IFN-λ secretion. Across models, ZIKV infection induced maternal IFN-λ2/3 responses (Fig. 4B). Importantly, maternal IFN-λ2/3 levels did not consistently correlate with viral titers and virus presence at the experimental endpoint (E16.5) (Fig. 4A and B). IFN-λ2/3 levels and viral titers were determined at 7 days post-infection (dpi) in Ifnar1 antibody-treated dams (infection models 1 and 2) and 9 dpi in immunocompetent dams (infection model 3), time points that capture established antiviral responses rather than acute induction. This dissociation between IFN-λ2/3 levels and viral titers was most evident following intravaginal infection, a route known to exhibit delayed antiviral signaling in the vaginal mucosa (37). This delayed response may sustain IFN-λ2/3 production even after viral clearance, with detectable IFN-λ2/3 persisting in 75% (3/4) of TNT-competent ZIKV-infected dams and 50% (2/4) of ZIKV^ΔTNT^-infected dams (Fig. 4A and B, model 2). IFN-λ2/3 levels were below the ELISA limit of detection in most immunocompetent dams. Although these values should not be interpreted as precise quantitative measurements, extrapolated concentrations were numerically higher after TNT-competent ZIKV infection than after ZIKV^ΔTNT^ infection in model 3 (12.9 vs 2.7; mean difference, 10.3; 95% CI, −0.50 to 21.0; *P* = 0.059) (Fig. 4B).

Strikingly, elevated maternal IFN-λ2/3 levels co-occurred with enhanced viral dissemination exclusively in dams infected subcutaneously with TNT-competent ZIKV, with detectable IFN-λ2/3 found in 100% of TNT-competent ZIKV-infected dams (3/3) and 0% of ZIKV^ΔTNT^-infected dams (0/3) (Fig. 4A and B, model 1). These findings suggest that TNT-forming capacity enables ZIKV to persist and disseminate *in vivo* despite activation of maternal IFN-λ2/3 responses, whereas loss of TNT-forming capacity of ZIKV^ΔTNT^ limits viral persistence and dissemination.

## DISCUSSION

Zika virus is uniquely capable among *Orthoflaviviruses* of vertical transmission in humans, yet the cellular mechanisms that enable placental dissemination have remained incompletely understood. Here, we identify tunneling nanotubes as a critical mechanism by which ZIKV infects placental cells *in vivo*. Using a TNT-deficient ZIKV mutant and complementary pregnancy models, we demonstrate that TNTs promote ZIKV infection of maternal and fetal tissues, drive placental pathology, contribute to fetal growth restriction, and have minimal impact on maternal disease. Mechanistically, TNT formation enables ZIKV to persist despite robust maternal IFN-λ responses, revealing a mode of immune evasion that is not captured by traditional models of cell-free viral transmission. These findings define TNT-mediated intercellular connectivity as a key determinant of ZIKV dissemination and pathogenesis during pregnancy.

Emerging evidence shows that viruses utilize TNTs to infect permissive and non-permissive cells, evade host antiviral defenses, and escape antiviral therapies (18, 38–43). Although TNT-like structures have been observed *in vivo* in HIV-infected mice (44), their functional contribution to viral dissemination has remained unresolved due to the lack of TNT-specific perturbation strategies. Here, using a TNT-deficient ZIKV mutant, we provide causal *in vivo* evidence that TNTs contribute to viral dissemination and pathogenesis during pregnancy. Pregnant dams infected with either wild-type ZIKV or ZIKV^ΔTNT^ did not develop overt clinical disease consistent with previous studies (45, 46) yet exhibited placental and fetal pathology. Importantly, loss of TNT-forming capacity in ZIKV^ΔTNT^ resulted in milder placental pathology following subcutaneous infection in Ifnar1 antibody-treated dams at E9.5. However, this effect was not observed upon intravaginal infection at E9.5 in dams also treated with Ifnar1 antibody, resulting in comparable pathology between ZIKV and ZIKV^ΔTNT^. In contrast, differences were evident in immunocompetent dams infected at E4.5–E7.5 via the same route (i.e., intravaginal), suggesting that TNT-dependent effects are shaped by both immune status and gestational timing.

Importantly, experimental modulation of immune status at either stage would likely result in the absence of infection or increased viral burden, complicating interpretation of placental and fetal outcomes. These findings should therefore be interpreted within the specific developmental windows examined. In mice, E4.5–E7.5 corresponds to early embryonic development and decidualization, whereas E9.5 represents late placentation (47). Developmentally, these stages broadly model the first trimester in humans (47), a period of increased susceptibility to ZIKV-associated fetal adverse outcomes (48, 49). Together, these findings suggest that TNT-mediated viral dissemination is context-dependent, with its contribution to placental pathology modulated by both host immunity and developmental stage.

In both subcutaneous and intravaginal infection models (Ifnar1 antibody treatment), ZIKV^ΔTNT^ failed to establish productive infection and efficiently disseminate to maternal and fetal tissues, in contrast to TNT-competent ZIKV. These findings suggest that TNT-forming capacity enhances viral dissemination across distinct routes of infection in the absence of IFN signaling. We suggest that differences in the extent of viral dissemination in maternal and fetal tissues following ZIKV infection reflect route-dependent effects on infection efficiency linked to TNT-forming capacity. Subcutaneous infection mimics mosquito-borne transmission and drives rapid systemic dissemination, whereas intravaginal infection models sexual transmission and is initially constrained by mucosal immunity in the female reproductive tract (50, 51). Consistent with this, immunocompetent dams infected intravaginally (model 3) failed to establish persistent infection by the experimental endpoint, consistent with IFN-mediated inhibition of viral replication (52). Nevertheless, observed placental and fetal pathology in ZIKV^ΔTNT^-infected dams suggests that, even under conditions restrictive to viral replication, TNT-forming capacity contributes to ZIKV pathogenesis. Although we could not assess transient viremia between infection and the experimental endpoint in this model, the absence of detectable viruses in maternal and fetal tissues does not exclude its occurrence. Supporting this, prior studies have detected ZIKV NS1 protein in fetal brains following intravaginal infection of immunocompetent dams at E4.5 or E8.5, consistent with transient viral dissemination (8).

Our *in vivo* findings do not fully recapitulate our prior *in vitro* observations that ZIKV exploits TNTs to evade antiviral signaling, such that loss of TNT-forming capacity results in heightened IFN-λ responses and viral clearance (18). Instead, we observed lower plasma IFN-λ levels in Ifnar1 antibody-treated dams infected subcutaneously with ZIKV^ΔTNT^ compared with ZIKV. One possible explanation is that an earlier robust IFN-λ response may have contributed to restricting ZIKV^ΔTNT^ replication following infection, but this could not be detected in our study design. Interestingly, we observed similarly detectable IFN-λ levels in Ifnar1 antibody-treated dams infected intravaginally with either ZIKV or ZIKV^ΔTNT^, despite evidence of viral clearance in the ZIKV^ΔTNT^ group. Given the reported delay in IFN signaling in the vaginal mucosa following ZIKV infection (37), the route of infection likely shapes the kinetics and detectability of these responses. This difference may provide a temporal window in the intravaginal model to capture residual IFN responses, particularly in the context of TNT-deficient ZIKV, while the rapid kinetics following subcutaneous infection may obscure transient IFN responses at the experimental endpoint. Although both IFN-λ (17, 53) and type I IFN (54) signaling have been linked to adverse pregnancy outcomes, IFN responses alone are unlikely to explain the placental and fetal pathology observed. Exploratory analyses showed that both viruses cause pathogenesis compared with uninfected controls, supporting a model in which TNT-forming capacity exacerbates ZIKV-associated pathogenesis rather than acting as its sole determinant.

Although TNTs and TNT-like structures have been visualized in various tissues using confocal microscopy and genetic labeling approaches (33), definitive identification of TNTs in placental tissue remains challenging because validated placenta-specific TNT markers are lacking and these structures are dynamic and fragile. Thus, limitations of our study include lack of TNT-specific inhibitors and TNT markers. First, we could not determine whether the observed effects were specifically due to the loss of TNT-mediated placental infection or resulted from the reduced systemic dissemination of the TNT-deficient virus. Although TNT formation can be inhibited by F-actin-disrupting agents such as latrunculin and cytochalasin D (55, 56), their broad effects on cellular processes and associated toxicity preclude their use in clinical settings. Second, while we observed TNT-like structures in ZIKV-infected placentas within both the junctional and labyrinth zones, we could not definitively identify these structures as TNTs because validated TNT-specific markers are lacking in placental tissue. Markers such as TTYH1 and GAP43 have been described in neural and breast tissues but are absent or expressed at low levels in the placenta (30, 57–59), suggesting that placental TNTs may be regulated by distinct molecular mechanisms. Furthermore, proteins associated with TNT formation, including F-actin and M-Sec (60), are ubiquitously expressed and not specific to TNTs, making it difficult to distinguish these structures within the dense placental architecture. Advances in TNT-specific markers and high-resolution imaging technologies may facilitate the visualization and characterization of TNTs *in vivo* (31, 61). Finally, although TNT-competent ZIKV and ZIKV^ΔTNT^ produced distinct placental and fetal outcomes, ZIKV^ΔTNT^ was not detected in placental tissue under the conditions tested. Therefore, we cannot determine whether the reduced placental infection and fetal pathology observed with ZIKV^ΔTNT^ reflect impaired systemic dissemination, reduced placental invasion, decreased TNT-mediated cell-to-cell spread within the placenta, or a combination of these mechanisms.

Together, our findings suggest that TNTs are co-opted by ZIKV to enhance maternal-fetal transmission and drive placental and fetal pathology *in vivo*. Across multiple infection models, TNT-forming capacity promotes viral dissemination and persistence, including under conditions that otherwise restrict infection, and is shaped by immune status, gestational timing, and route of infection. These data suggest a model in which TNT enables ZIKV to disseminate to the placenta despite antiviral responses, including IFN-λ signaling. Future studies should define whether TNTs facilitate immune evasion at earlier time points following ZIKV infection and therapeutic resistance, and whether targeting TNT biogenesis can limit viral pathogenesis. In addition, further investigation of the role of TNTs in systemic viral dissemination beyond the placenta may provide important insights into ZIKV disease progression.

## MATERIALS AND METHODS

### Viruses and cells

The African ZIKV MR-766 (Uganda) strain was obtained from BEI Resources. The infectious and replication-competent ZIKV∆TNT mutant was previously generated by our group by introducing amino acid substitutions into the ZIKV MR-766 strain cDNA clone (18). Vero E6 cells (ATCC, #CRL-1586) were cultured in Dulbecco’s modified Eagle medium/nutrient mixture F-12 (DMEM/F-12; Gibco, #11330032) supplemented with 10% fetal bovine serum (FBS) (Gibco, #16140071) and maintained at 37°C with 5% CO_2_.

### Zika virus propagation and titration

Confluent Vero E6 cell monolayers were infected with ZIKV and maintained for five days in phenol red-free DMEM/F-12 (Gibco, #21041025) supplemented with 10% FBS. At 2, 3, 4, and 5 days post-infection, virus-containing supernatants were harvested, supplemented with 25 mM HEPES (Gibco, #15630080), and stored at 4°C. Collected supernatants were pooled, clarified by centrifugation at 3,000 × g for 5 min, and subsequently filtered through a 0.45 μm PVDF syringe filter. The virus stock was then concentrated using Amicon Ultra Centrifugal Filter (Sigma-Aldrich, #UFC9100) according to the manufacturer’s protocol. Concentrated virus was aliquoted and stored long-term at −80°C. A frozen aliquot was used for determining viral titers for infection by plaque assay of serial dilutions on Vero E6 monolayers (7). To determine virus titer in mouse tissues, samples were thawed, weighed, and homogenized with zirconia beads (BioSpec products, #11079110zx) in a TissueLyser instrument (Qiagen) equipped with cold adaptor plates at 30 Hz for 10 min. Then, 200 μL of 2% FBS DMEM/F12 was added to the lysed tissue. Samples were clarified by centrifugation (2,000 × g at 4°C for 10 min), and 10-fold serial dilutions were prepared, and viral titer was determined by plaque assay on Vero E6 cells. Samples with no detectable plaques (0 PFU) were assigned a value of 1 PFU/g prior to log10 transformation for visualization purposes.

### Mice

Immunocompetent outbred CD-1 mice (2–4 months of age) were purchased from Charles River Laboratories and maintained at Baylor College of Medicine in a biosafety level two facility (ABSL2) with *ad libitum* access to food and water. Standard rodent chows and water were provided *ad libitum* throughout the experiment. Mice were housed in groups of four in a temperature (22 ± 1°C) and humidity-controlled vivarium under a 12:12 light/dark cycle. Mating was set at 1:2 male to female ratio, and pregnancy was confirmed by checking for the presence of a vaginal plug the following morning and determined as gestational age E0.5. All mice were humanely euthanized at the end of each experiment.

### *In vivo* infections

Infection via the intravaginal route was adapted from Yockey et al. (8). Briefly, immunocompetent dams were intravaginally inoculated with viruses on E4.5 to E7.5 at the same time across days and under continuous anesthesia with isoflurane. By using a pipet, 20 μL of ZIKV (MR-766 and ZIKV^ΔTNT^), equivalent to 1.5 × 10^6^ PFU, were inoculated in the vaginal lumen with a 15–20-min inoculation. For subcutaneous and intravaginal infection, type I IFN was transiently suppressed by intraperitoneal injection of 2.5 mg monoclonal anti-mouse Ifnar1 blocking antibody (MAR1-5A3, Leinco, #l-401) at E8.5 the day before infection. At E9.5, dams were infected with 1 × 10⁴ PFU (subcutaneously) or 1.7 × 10⁴ PFU (intravaginally). All dams were monitored daily until euthanasia at E16.5.

### Tissue harvesting

On E16.5, pregnant dams were euthanized. Blood was collected, and plasma was isolated by centrifugation for subsequent interferon analysis. Dams were perfused with PBS, and the uterine horns were collected and carefully opened to release the fetoplacental units. The spleen, uterus, placentas, and fetuses were harvested and weighed. Tissues were fixed in 4% PFA overnight, and the next day, tissues were processed for histology. Fetal OF length, crown-to-rump length (CRL), and placental diameter were measured. Placental efficiency was calculated as the ratio of fetal weight to placental weight. Fetal size was calculated as OF × CRL. Fragments of each tissue were collected and stored at −80°C for subsequent determination of viral titers.

### ELISA

Mouse plasma IFN-λ2/3 levels were measured using the Mouse IL-28A/B (IFN-λ2/3) DuoSet ELISA Kit (R&D Systems, #DY1789B) according to the manufacturer’s instructions. Absorbance was read at 490 nm using a BioTek Epoch microplate reader, and concentrations were calculated accordingly. The assay limit of detection (LOD) was 43.71 pg/mL.

### Hematoxylin and eosin staining and analysis

Mouse placenta paraffin tissue sections (5 µm) were deparaffinized using Histo-Clear solution (Electron Microscopy Sciences, #64110-04) and rehydrated through a graded ethanol series (100%, 95%, 90%, 70%, and 50%), followed by distilled water. Sections were stained with hematoxylin (Epredia, #7211) and eosin (Epredia, #71204). Whole-placenta sections were scanned using a Pannoramic MIDI III Digital Scanner (3DHISTECH) with a 20× objective. The total placental area, as well as the areas of the junctional zone and labyrinth zone, was manually outlined and automatically quantified using the SlideViewer software (3DHISTECH) by a blinded investigator.

### Immunohistochemistry and immunofluorescence assays

For immunohistochemistry, deparaffinized and rehydrated placenta sections underwent antigen retrieval in a 10 mM sodium citrate buffer (pH 6.0) using a pressure cooker for 2 min. Slides were washed in high salt PBS (300 mM NaCl, Sigma-Aldrich, #746398) and stored overnight at 4°C. Autofluorescence was initially quenched by incubation with 0.1% (wt/vol) Sudan Black B (Fisher Scientific, #ICN15208825). Sections were then blocked for 45 min at room temperature in the blocking buffer (5% normal goat serum, 0.1% Tween-20, and 1% BSA in high salt PBS). Sections were incubated with primary antibodies, including 1:500 rabbit anti-NS1 (GeneTex, #GTX133323), 1:200 chicken anti-TOMM20 (Antibodies Inc., #TOMM20-0100), and 3:188 rat anti-CK8 (DSHB, #TROMA-I), diluted in antibody dilution buffer (1% normal goat serum, 1% BSA, and 0.1% Tween-20 in high salt PBS) for 1 h at room temperature. Alexa Fluor-conjugated secondary antibodies (1:500), including anti-rabbit 488 (Thermo Fisher, #A-11034), anti-rat 594 (Thermo Fisher, #A-11007), and anti-chicken 647 (Thermo Fisher, #A-21449), were applied for 1 h at room temperature. Slides were further quenched with Vector TrueVIEW Autofluorescence Quenching Kit (Vector Laboratories, #SP-8400-15) according to the manufacturer’s protocol and counterstained with 1:10,000 Hoechst 33342 (Thermo Fisher, #H3570). Sections were mounted using ProLong Glass Antifade Mountant (Thermo Fisher, #P36980) and imaged using a Nikon A1R-MP confocal microscope fitted with a 40× objective lens with 1.4 numerical aperture and processed using ImageJ.

### Statistical analysis

All analyses were performed using GraphPad Prism 9. Outliers were assessed using Grubbs’ test. Statistical analyses were selected based on data distribution and structure. For the primary hypothesis, an unpaired *t*-test with Welch’s correction was used to compare infection outcomes between TNT-competent ZIKV and ZIKV^ΔTNT^-infected groups. Comparisons with uninfected controls were considered secondary analyses and were analyzed using Welch’s one-way ANOVA followed by Dunnett’s T3 multiple-comparison test. All tests were two-sided, and *P*-value  <  0.05 was considered statistically significant. Data are presented as mean ± SEM for histograms and as median with interquartile range (25th–75th percentiles) for violin plots.

## Data Availability

All data generated or analyzed during this study are included in the article and its supplemental materials.

## SUPPLEMENTAL MATERIAL

Fig. S1 mbio.01562-26-s0001.pdf TNT-competent ZIKV infection of placentas across models.

Supplemental material legends mbio.01562-26-s0002.docx Fig. S1 and Table S1 legends.

Table S1 mbio.01562-26-s0003.pdf Secondary analyses of maternal and fetal outcomes across infection models.

ASM does not own the copyrights to Supplemental Material that may be linked to, or accessed through, an article. The authors have granted ASM a non-exclusive, world-wide license to publish the Supplemental Material files. Please contact the corresponding author directly for reuse.
