# Supplementary material for: Tunneling nanotubes contribute to Zika virus pathogenesis at the maternal-fetal interface in vivo
Source: mBio. 2026 Aug 21;17(9):e01562-26. doi: 10.1128/mbio.01562-26 (PMC13556233; doi:10.1128/mbio.01562-26)
Supplement: Supplementary file 1 [file mbio.01562-26-s0001.pdf]

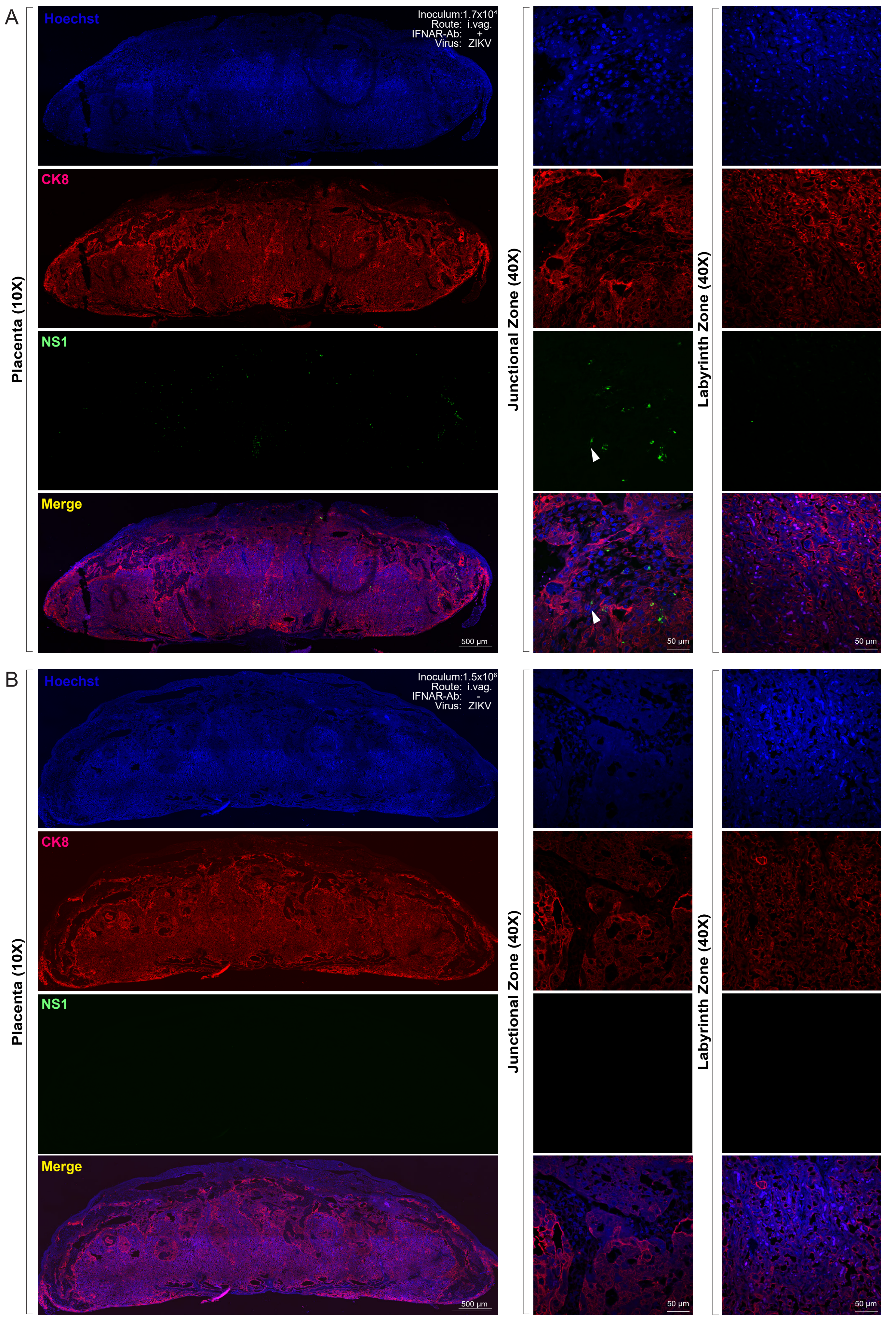

A
Hoechst
Inoculum:1.7x10^4
Route: i.vag.
IFNAR-Ab: +
Virus: ZIKV
CK8
NS1
Merge
Placenta (10X)
Junctional Zone (40X)
Labyrinth Zone (40X)
500 µm
50 µm
50 µm
B
Hoechst
Inoculum:1.5x10^6
Route: i.vag.
IFNAR-Ab: -
Virus: ZIKV
CK8
NS1
Merge
Placenta (10X)
Junctional Zone (40X)
Labyrinth Zone (40X)
500 µm
50 µm
50 µm
