# Supplementary material for: Tunneling nanotubes contribute to Zika virus pathogenesis at the maternal-fetal interface in vivo
Source: mBio. 2026 Aug 21;17(9):e01562-26. doi: 10.1128/mbio.01562-26 (PMC13556233; doi:10.1128/mbio.01562-26)
Supplement: Supplementary file 2 [file mbio.01562-26-s0003.pdf]

Table S1. Secondary analyses comparing maternal and fetal outcomes in dams infected with TNT-competent ZIKV or ZIKV$^{\Delta TNT}$ relative to uninfected controls across infection models.

Variables	Infection model 1					Infection model 2					Infection model 3				
	**Mock[a]**	**MR-766[b]**	**ZIKV$^{\Delta TNT}$ [c]**	**P-value a,b**	**P-value a,c**	**Mock[d]**	**MR-766[e]**	**ZIKV$^{\Delta TNT}$ [f]**	**P-value d,e**	**P-value d,f**	**Mock [g]**	**MR-766[h]**	**ZIKV$^{\Delta TNT}$ [i]**	**P-value g,h**	**P-value g,i**
**Dam weight (g), mean ± SEM (n)**	53.2 ± 3.6 (n=2)	54.13 ± 1.9 (n=3)	55.7 ± 3.1 (n=3)	0.972	0.856	53.9 ± 3.4 (n=2)	49.88 ± 2.7 (n=4)	49.7 ± 2.1 (n=4)	0.651	0.590	52.2 ± 2.0 (n=5)	52.6 ± 1.7 (n=8)	53.6 ± 2.6 (n=8)	0.983	0.890
**Spleen weight (mg), mean ± SEM (n)**	267.9 ± 20.7 (n=2)	388.0 ± 38.4 (n=3)	262.2 ± 32.1 (n=3)	0.121	0.986	174.5 ± 41.5 (n=2)	309.4 ± 32.3 (n=4)	177.1 ± 19.3 (n=3)	0.198	0.998	294.6 ± 78.4 (n=4)	208.0 ± 12.6 (n=8)	187.1 ± 18.5 (n=7)	0.547	0.436
**Litter size, mean ± SEM (n)**	13.5 ± 1.5 (n=2)	14.7 ± 0.9 (n=3)	14.3 ± 0.9 (n=3)	0.079	0.873	13.0 ± 3.0 (n=2)	12.3 ± 1.7 (n=4)	11.8 ± 0.5 (n=4)	0.970	0.907	13.2 ± 0.6	13.2 ± 0.5	13.2 ± 0.9	0.998	0.999
**Placental weight (mg), mean ± SEM (n)**	128.8 ± 3.8 (n=28)	125.6 ± 2.4 (n=43)	117.5 ± 2.5 (n=41)	0.737	**0.034**	114.4 ± 2.9 (n=29)	110.9 ± 2.8 (n=48)	114.4 ± 2.8 (n=46)	0.616	1.000	120.3 ± 2.8 (n=36)	131.1 ± 2.4 (n=82)	128.0 ± 2.2 (n=65)	**0.010**	0.073
**Placental diameter (mm), mean ± SEM (n)**	7.6 ± 0.1 (n=27)	8.37 ± 0.04 (n=43)	7.76 ± 0.1 (n=39)	**<0.0001**	0.090	8.0 ± 0.1 (n=29)	7.8 ± 0.1 (n=40)	7.9 ± 0.1 (n=27)	0.236	0.604	8.3 ± 0.1 (n=38)	8.3 ± 0.1 (n=82)	8.4 ± 0.1 (n=65)	0.669	0.747
**JZ/LZ ratio, mean ± SEM (n)**	–	0.8 ± 0.1 (n=7)	0.5 ± 0.1 (n=6)	n.a	n.a	0.5 ± 0.04 (n=6)	0.4 ± 0.1 (n=3)	0.3 ± 0.1 (n=3)	0.755	0.193	0.6 ± 0.03 (n=36)	0.5 ± 0.01 (n=57)	0.6 ± 0.02 (n=54)	0.371	0.867
**Placental efficiency, mean ± SEM (n)**	4.6 ± 0.2 (n=26)	5.1 ± 0.1 (n=44)	5.6 ± 0.2 (n=41)	**0.014**	**0.001**	5.6 ± 0.1 (n=26)	6.1 ± 0.1 (n=47)	6.2 ± 0.1 (n=46)	0.102	0.021	4.9 ± 0.1 (n=38)	5.0 ± 0.1 (n=81)	5.4 ± 0.1 (n=66)	0.931	**0.036**
**Fetal Weight (mg), mean ± SEM (n)**	595.9 ± 17.5 (n=26)	639.4 ± 7.1 (n=44)	665.5 ± 10.3 (n=42)	0.055	**0.003**	650.9 ± 16.3 (n=26)	669.6 ± 12.6 (n=48)	693.5 ± 11.1 (n=47)	0.600	0.069	593.4 ± 11.7 (n=38)	651.1 ± 9.8 (n=80)	694.2 ± 8.9 (n=64)	**0.001**	**<0.0001**
**Fetal OF (mm), mean ± SEM (n)**	6.4 ± 0.1 (n=26)	6.7 ± 0.04 (n=44)	6.9 ± 0.03 (n=41)	**<0.0001**	**<0.0001**	7.0 ± 0.1 (n=26)	7.3 ± 0.04 (n=47)	7.4 ± 0.03 (n=46)	**0.004**	**<0.0001**	7.2 ± 0.1 (n=38)	7.0 ± 0.1 (n=80)	7.4 ± 0.1 (n=66)	**0.013**	0.1079
**Fetal CRL (mm), mean ± SEM (n)**	15.0 ± 0.1 (n=26)	15.4 ± 0.1 (n=44)	15.7 ± 0.1 (n=42)	**0.026**	**0.0003**	15.5 ± 0.2 (n=26)	16.1 ± 0.1 (n=48)	16.3 ± 0.1 (n=47)	**0.015**	**0.002**	16.5 ± 0.1 (n=38)	16.8 ± 0.1 (n=81)	17.6 ± 0.1 (n=66)	0.224	**<0.0001**
**Fetus size (mm^2), mean ± SEM (n)**	96.2 ± 1.4 (n=26)	103.7 ± 1.1 (n=44)	107.8 ± 1.5 (n=42)	**0.0003**	**<0.0001**	109.7 ± 2.1 (n=26)	118.5 ± 1.6 (n=48)	120.8 ± 1.6 (n=47)	**0.003**	**<0.0001**	119.6 ± 1.8 (n=38)	118.7 ± 1.6 (n=80)	129.8 ± 1.7 (n=66)	0.900	**0.0002**
**IFN-λ2/3 >LOD, n/N (%)**	0/2 (0%)	3/3 (100%)	0/3 (0%)	n.a	n.a	0/2 (0%)	3/4 (75%)	2/4 (50%)	na	Na	0/3 (0%)	0/8 (0%)	0/8 (0%)	n.a	n.a

Fetal and maternal outcomes at experimental endpoint at E16.5. Infection model 1: Anti-IFNAR treated dams (E8.5) were infected subcutaneously ($1x10^4$ PFUs) with TNT-competent ZIKV (MR-766), TNT-deficient ZIKV (ZIKV$^{\Delta TNT}$), or mock infected (Mock). Infection model 2: Anti-IFNAR treated dams (E8.5) were infected intravaginally ($1.7x10^4$ PFUs) with TNT-competent ZIKV (MR-766), TNT-deficient ZIKV (ZIKV$^{\Delta TNT}$), or mock infected (Mock). Infection model 3: Immunocompetent dams (E8.5) were infected intravaginally (E4.5-E7.5; $1x10^6$ PFUs) with TNT-competent ZIKV (MR-766), TNT-deficient ZIKV (ZIKV$^{\Delta TNT}$), or mock infected (Mock). SEM, standard error of the mean. g, grams. mg, milligrams. mm, millimeter. IFN, interferon. LOD, limit of detection. n, number of observations. N, total number of observations. n.a, not applicable. –, data not available. For all comparisons, Welch's one-way ANOVA followed by Dunnett's T3 multiple-comparison test was used. A two-sided P-value <0.05 was considered statistically significant.
