# Supplementary material for: Tunneling nanotubes contribute to Zika virus pathogenesis at the maternal-fetal interface in vivo
Source: mBio. 2026 Aug 21;17(9):e01562-26. doi: 10.1128/mbio.01562-26 (PMC13556233; doi:10.1128/mbio.01562-26)
Supplement: Supplementary file 3 [file mbio.01562-26-s0002.docx]

**Supplementary material**

**Figure S1. TNT-competent ZIKV infection of placentas across models.** Representative immunofluorescence images of E16.5 placentas from pregnant dams infected with TNT-competent ZIKV. In Ifnar1 antibody–treated (IFNAR-Ab) dams infected intravaginally at E9.5, ZIKV NS1 localized to both the junctional and labyrinth zones. In contrast, placentas from immunocompetent dams infected via the same route at E4.5–E7.5 showed no detectable NS1. Placentas were stained for ZIKV NS1 (green), cytokeratin 8 (CK8; red), and nuclei (Hoechst, blue). White arrows indicate NS1 signal. Large-area images were acquired using a 10× confocal objective (scale bar, 500 µm); insets show representative 40× images of the junctional and labyrinth zones (scale bar, 25 µm). i.vag, intravaginal; s.c., subcutaneous.

**Table S1. Secondary analyses comparing maternal and fetal outcomes in dams infected with TNT-competent ZIKV or ZIKVΔTNT relative to uninfected controls across infection models.**
